# Prevention of Rebleeding in Colonic Diverticular Bleeding Using Orengedokuto: Protocol for a Double-Blind Randomized Controlled Trial

**DOI:** 10.2196/93449

**Published:** 2026-09-22

**Authors:** Takashi Hisabe, Ken Kinjo, Keiko Ogawa-Ochiai, Keita Mori, Ryosuke Amano, Toshihiro Sakurai, Suketo Sou, Kazuhiro Takeda, Rino Hasegawa, Hideki Ishikawa

**Affiliations:** 1 Department of Gastroenterology, Fukuoka University Chikushi Hospital, 1-1-1 Zokumyoin, Chikushino, Fukuoka, 818-8502, Japan, 81 570-02-777; 2 Department of Gastroenterology, Urasoe General Hospital, Urasoe, Japan; 3 Kampo Clinical Center, Hiroshima University Hospital, Hiroshima, Japan; 4 Department of Biostatistics, Shizuoka Cancer Center, Suntogun, Japan; 5 Department of Gastroenterology, Ashiya Central Hospital, Ongagunn, Japan; 6 Department of Gastroenterology, Tobata Kyoritsu Hospital, Kitakyusyu, Japan; 7 Department of Gastroenterology, Tagawa Municipal Hospital, Tagawa, Japan; 8 Department of Gastroenterology, Sada Hospital, Fukuoka, Japan; 9 Department of Molecular-Targeting Prevention, Kyoto Prefectural University of Medicine, Kyoto, Japan

**Keywords:** Kampo, orengedokuto, colonic diverticular bleeding, hemostasis, randomized controlled trial, rebleeding, lower gastrointestinal bleeding, colonoscopy, endoscopy, study protocol

## Abstract

**Background:**

Endoscopic hemostasis is used to treat colonic diverticular bleeding (CDB); however, rebleeding is frequently observed after conservative treatment. Currently, evidence regarding the efficacy of oral medications for preventing rebleeding from colonic diverticula is limited. Orengedokuto (Huanglian Jiedu Tang) is a traditional Kampo formula with capillary-constricting and hemostatic properties that are used to treat bleeding conditions such as epistaxis, hematemesis, and melena. If proven to be effective in preventing rebleeding, orengedokuto could reduce the need for hospitalization and repeat endoscopic procedures, thereby lowering the burden on patients and health care systems.

**Objective:**

This study aims to exploratorily evaluate the efficacy and safety of orengedokuto in preventing rebleeding in patients with CDB who have achieved spontaneous hemostasis.

**Methods:**

We designed a double-blind, randomized controlled trial in patients with CDB who achieved spontaneous hemostasis and in whom the bleeding source could not be identified, making endoscopic hemostasis unfeasible. Patient allocation will be determined using a dynamic randomization method based on minimization, with a 1:1 assignment ratio. Treatment will begin within 24 hours of enrollment. The participants will receive either orengedokuto plus standard treatment or placebo plus standard treatment for 14 days, followed by a 31-day observation period. They will take 2 capsules of either orengedokuto or placebo 3 times a day. Standard treatment refers to the standard approach for managing CDB, which involves resting the bowel through fasting and intravenous fluid administration. Corn starch will be used for the placebo. The primary end point is the proportion of patients who experience rebleeding. Secondary end points include the length of hospital stay, time to resumption of oral intake, severity of rebleeding (including the timing of rebleeding and degree of anemia), and incidence of secondary rebleeding. Safety will be assessed through monitoring of adverse events for causality and frequency. For the primary end point, the full analysis set will be used as the analysis population. The proportion of patients with rebleeding will be calculated for each group, and intergroup comparisons will be performed. For the secondary end points, statistical comparisons will be performed between the 2 groups.

**Results:**

This study received funding from the Japan Agency for Medical Research and Development in May 2025. Enrollment is scheduled from July 2025 to December 2027. As of June 2026, we have enrolled 48 participants. All follow-up and data collection activities will be finalized by February 2028. Results are anticipated to be completed in the first quarter of 2028.

**Conclusions:**

If orengedokuto demonstrates a hemostatic effect, it could offer a less invasive alternative to endoscopic hemostasis, potentially reducing hospital stays and overall health care resource use.

## Introduction

The number of patients with conditions that cause acute lower gastrointestinal bleeding (ALGIB) is increasing [1-3]. Recently, colonic diverticular bleeding (CDB) has been reported as the most common cause of ALGIB in Japan [4-6]. The incidence of CDB has increased since 2000, and the number of patients with severe CDB has increased proportionately [3,7]. Endoscopic hemostasis therapy in patients with high-risk stigmata of recent hemorrhage (SRH), including active bleeding, nonbleeding visible vessels, and adherent clots, can prevent recurrent bleeding and decrease the need for surgery [8].

Endoscopic treatment is recommended when active bleeding or SRH occurs, irrespective of etiology [9,10]. Colonoscopy can be used to identify SRH, and endoscopic treatment can suppress rebleeding [8,11-16]. However, SRH is observed in only 8% to 15% of CDB cases [17-19]. In several instances, bleeding stops by the time of colonoscopy; moreover, owing to the presence of numerous diverticula, the rate of identifying the bleeding source is not high. When conservative treatment is administered without endoscopic hemostasis in patients showing signs of bleeding, the early rebleeding rate within 1 month is high (53%‐66%) [8,20].

There is no evidence regarding the effectiveness of pharmacological treatments for hemostasis in cases of diverticular bleeding in which endoscopic hemostasis cannot be performed.

Traditional herbal medicines have long been used to treat various diseases, particularly in Asian countries.

Orengedokuto (Huanglian Jiedu Tang) is a traditional Kampo formula originating from classical Chinese medicine and was first recorded in the ancient medical text Wai Tai Mi Yao [21]. The formula consists of 4 medicinal herbs: Coptidis Rhizoma, Scutellariae Radix, Phellodendri Cortex, and Gardeniae Fructus. Orengedokuto has a long history of use in both Traditional Chinese Medicine and Japanese Kampo medicine to treat heat toxin syndromes, inflammatory conditions, and irritability [21]. It remains the cornerstone formula for clearing internal heat [22]. In Japan, the indications covered by insurance include nosebleeds, hypertension, insomnia, gastritis, dizziness, and palpitations. Orengedokuto is suggested to be involved in primary hemostasis, with potential effects such as vasoconstriction and inhibition of the abnormal activation of the coagulation-fibrinolysis system. Although hemostatic effects on gastrointestinal bleeding are expected, to date, no reports have verified their efficacy. We conducted a retrospective study of patients diagnosed with CDB following colonoscopy at 5 institutions over a 1-year period (January to December 2019; UMIN000049020). We evaluated the rate of rebleeding within 30 days in patients who received conservative treatment because endoscopic hemostasis could not be performed, comparing a group that received orengedokuto with a group that did not. The rate of rebleeding within 30 days was 9.1% (1/11) in the orengedokuto group and 31.1% (15/48) in the control group. Although the difference was not statistically significant (*P*=.26), the rebleeding rate was lower in patients who received orengedokuto. Furthermore, in this study, cases of rebleeding occurred within 10 days.

We designed an exploratory double-blind randomized controlled trial to investigate the efficacy and safety of orengedokuto in preventing rebleeding in patients who experienced spontaneous hemostasis after CDB and in whom the bleeding source could not be identified, thus precluding endoscopic hemostasis. We hypothesize that adjunctive treatment with orengedokuto will reduce the incidence of rebleeding and improve clinical outcomes without compromising safety compared with placebo.

## Methods

### Study Design

In this double-blind, randomized controlled trial, patient allocation will be determined using a minimization-based dynamic randomization method. The stratification factors for allocation are as follows: (1) study site, (2) use of antithrombotic agents (yes/no), (3) history of CDB (yes/no), and (4) age at enrollment (≤69 y/≥70 y). Patients will be assigned in a 1:1 ratio to either the orengedokuto group (orengedokuto plus standard treatment) or the placebo group (placebo plus standard treatment).

**Figure 1.**
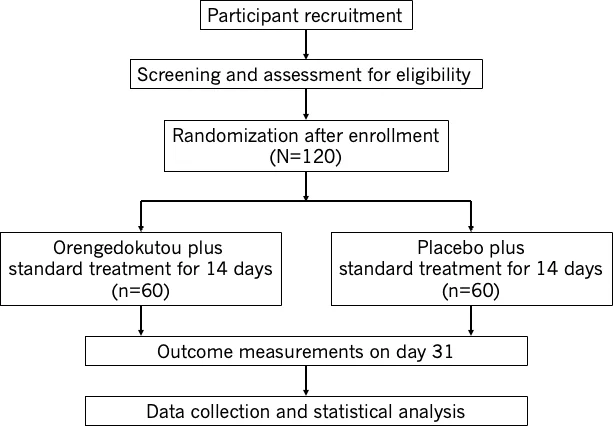
Study flowchart.

The study setting parallels usual practice. Patients will be recruited from the gastroenterology inpatient departments of hospitals in different districts of Japan. A SPIRIT (Standard Protocol Items: Recommendations for Interventional Trials) 2025 checklist is provided in Checklist 1. A flowchart of the study is shown in Figure 1.

### Ethical Considerations

The study protocol was reviewed and approved by the Institutional Review Board of Kyoto Prefectural University of Medicine (approval number CRB5200001). The trial was registered in the Japan Registry for Clinical Trials (jRCTs051250063) on July 8, 2025. All participants will receive comprehensive study information, including the study’s purpose, potential risks, benefits, and their right to withdraw at any time, and will be required to provide written informed consent before screening. All participants may withdraw from the study at any time without consequence. Participant confidentiality will be ensured by assigning anonymized identification codes. Data are being stored in secure, password-protected databases accessible only to the research team. No personal identifiers will be disclosed in any publication derived from this study. This study is being conducted in accordance with the principles outlined in the Declaration of Helsinki and the Ethical Guidelines for Medical and Biological Research Involving Human Subjects.

The principal investigator (PI) or coinvestigator will select potential participants, confirm that they meet the inclusion criteria and do not meet any of the exclusion criteria, provide written information to potential participants, and obtain written consent.

### Eligibility Criteria

#### Participants

The target condition is CDB with spontaneous hemostasis, where the bleeding source cannot be identified during endoscopy; hence, endoscopic hemostasis cannot be performed. Confirmed CDB was defined as the presence of SRH within a diverticulum on colonoscopy. Suspected CDB was defined as either contrast-enhanced computed tomography demonstrating contrast extravasation localized to a diverticulum without the subsequent endoscopic confirmation of SRH or the absence of SRH on colonoscopy, despite reasonable exclusion of other causes of lower gastrointestinal bleeding, making diverticular bleeding the most likely diagnosis. We set the inclusion criteria as a colonoscopy performed within 24 hours of the initial visit. The reason for this was that this study focused on patients with colonoscopically confirmed diverticular bleeding, and we prioritized the internal validity of the study by enhancing diagnostic accuracy.

Patients are required to meet all of the following inclusion criteria and none of the exclusion criteria.

#### Inclusion Criteria

The inclusion criteria are as follows:

1. Patients aged 18 to 89 years at the time of providing informed consent.
2. Patients who present to the hospital within 48 hours of the onset of hematochezia. A colonoscopy was performed within 24 hours of the initial visit, and the patient was diagnosed with diverticular bleeding; however, because no SRH (active bleeding, nonbleeding visible vessels, and an underlying adherent clot with active bleeding or nonbleeding visible vessels) was detected, endoscopic hemostasis could not be performed, and the bleeding has since stopped spontaneously.
3. Patients who experience dizziness or palpitations.
4. Hospitalized patients with a shock index [23] of less than 1.0.
5. Patients who have been adequately informed about the study, have fully understood the explanation, and have voluntarily provided written informed consent.

#### Exclusion Criteria

The exclusion criteria are as follows:

1. Patients in whom the responsible diverticulum could not be identified through colonoscopy and were scheduled to undergo emergency surgery, transarterial embolization, or barium enema therapy due to ongoing bleeding;
2. Patients with a history of colorectal resection (excluding appendectomy);
3. Patients who have received orengedokuto (Huanglian Jiedu Tang) within 2 weeks prior to colonoscopy;
4. Patients with a history of CDB within 30 days prior to colonoscopy;
5. Patients in whom the cecum could not be visualized during colonoscopy;
6. Patients with severe organ dysfunction defined as follows (laboratory tests within 4 weeks prior to treatment are acceptable):
  - Renal function: serum creatinine level 1.5 times more than the normal threshold for the institution;
  - Hepatic function: serum aspartate aminotransferase or alanine aminotransferase levels more than 3 times the normal threshold for the institution; and
  - Electrolytes: serum sodium level of less than 125 mEq/L or serum potassium level of less than 3.3 mEq/L.
7. Women who are pregnant or breastfeeding, who may be pregnant, or who are planning to conceive;
8. Patients with a history of allergy to orengedokuto;
9. Other causes of gastrointestinal bleeding identified during colonoscopy, such as advanced colorectal cancer, ulcerative colitis, Crohn's disease, or infectious colitis;
10. Patients who are currently participating in, or intend to participate, in another clinical trial during the study period; and
11. Any other patients whom the PI or subinvestigator deems unsuitable for participation in the study

### Interventions

Both groups will receive standard treatment (bowel rest through fasting and intravenous fluid administration), which is the standard treatment for CDB. When comparing the orengedokuto group with the placebo group, the only difference between the test groups will be the presence or absence of the active ingredient in the intervention being tested. There is no available treatment for CDB; hence, there is no inherent disadvantage of using a placebo. Moreover, the placebo effect can be controlled by using a placebo as the test drug for the placebo group.

The intervention will commence within 24 hours of registration. Based on the allocation results, patients will receive either orengedokuto (orengedokuto plus standard treatment) or placebo (placebo plus standard treatment) for 14 days. Patient registration will be conducted through a web-based registration and allocation system managed by the data center. An interactive web system will ensure that the allocation remains concealed until it is assigned. The allocation sequence will be computer-generated and allocated using the interactive web system. Investigators or subinvestigators at each participating site will access the registration system via its URL to complete registration and obtain allocation results.

Subsequently, an observational follow-up will be conducted for 31 days from the start of treatment. Standard care refers to fasting and bowel rest, which are routinely used to treat CDB.

Orengedokuto is a traditional herbal medicine used in Japan. Each dose (6 capsules) contains the following herbal ingredients: ogon (3.0 g), oren (1.5 g), sanshishi (2.0 g), and obaku (1.5 g). Regarding the dosage, in accordance with insurance coverage guidelines, the recommended regimen is 2 capsules 3 times daily. Therefore, patients will take 2 capsules of either orengedokuto or placebo 3 times a day. As our retrospective analysis found that cases of rebleeding occurred within 10 days, we have set the treatment duration to 14 days. This study is being conducted using the investigational drug, orengedokuto, and a placebo both provided free of charge by Kotaro Kampo Pharmaceutical Co, Ltd. The placebo contains corn starch as the inactive filling material and was manufactured and supplied by Kotaro Kampo Pharmaceutical Co, Ltd. The placebo capsules were designed to be indistinguishable from the active capsules in appearance, shape, labeling, packaging, and administration schedule.

**Figure 2.**
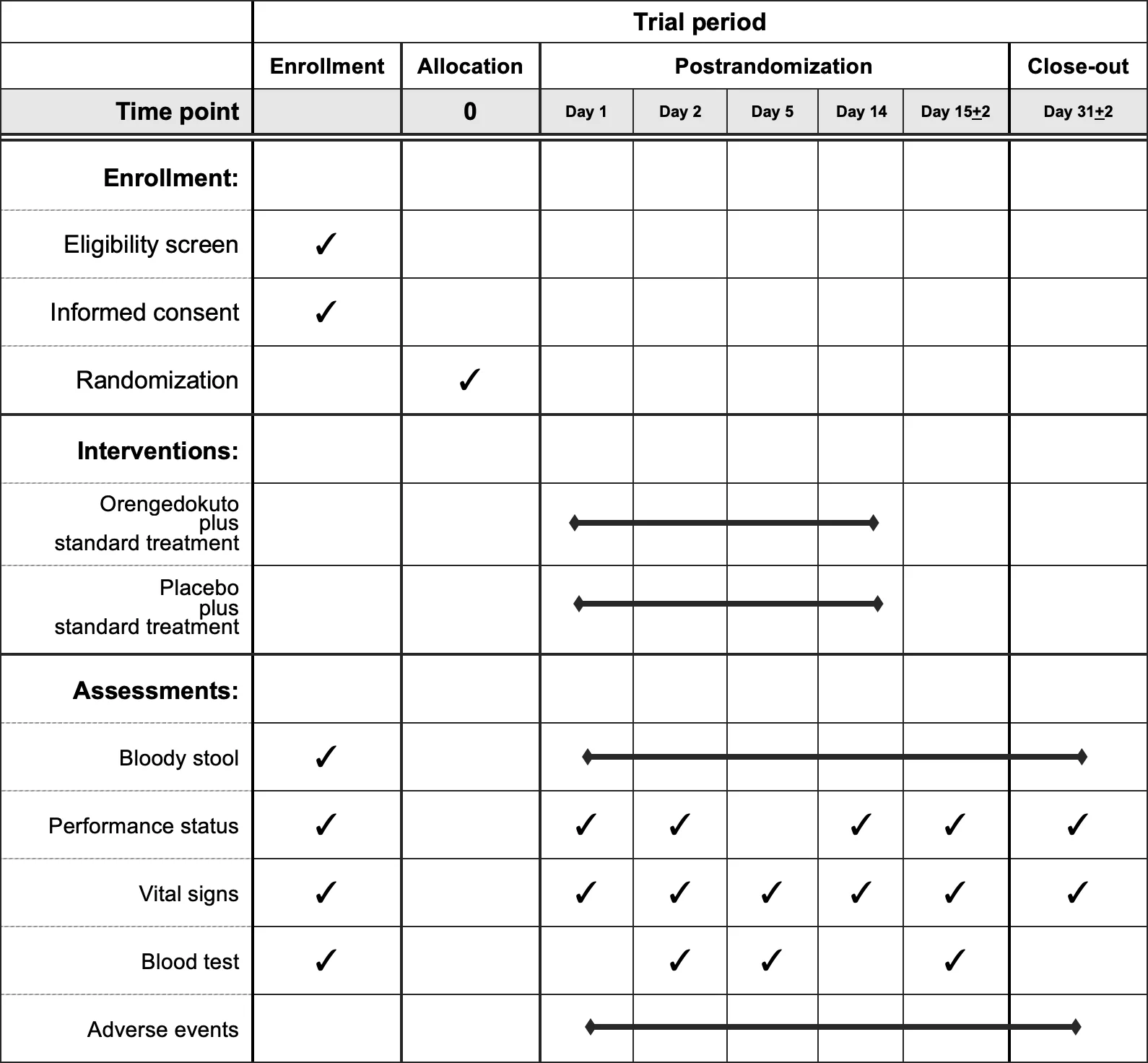
Schedule of enrollment, interventions, and assessments.

Blood tests, confirmation of hematochezia, and physical examinations will be performed prior to enrollment and on the day after the intervention. During hospitalization, daily assessments for hematochezia and physical examinations will be performed. On day 15 of the intervention, blood tests, confirmation of hematochezia, and physical examinations will be repeated. On day 31, confirmation of hematochezia and physical examinations will be conducted. Figure 2 shows the participants’ timelines.

All patients with ALGIB undergo colonoscopy for diagnosis and treatment within 24 hours of admission. Patients will be administered hypertonic polyethylene glycol solution (1‐2 L) for bowel preparation, except for those with contraindications. Patients with hemodynamic instability will be administered enemas.

### Criteria for Discontinuing or Modifying Allocated Interventions

The PI or subinvestigator shall discontinue the investigational drug and terminate the patient’s participation in the study if it is determined, for any of the following reasons, that continued participation is not feasible. In such cases, the reason for discontinuation will be explained to the study participants, as necessary. Furthermore, the postdiscontinuation treatment will be handled sincerely to avoid any disadvantages to the participant.

Discontinuation criteria are as follows:

1. If the participant withdraws consent to participate in the study;
2. If it is found after enrollment that the participant does not meet the eligibility criteria (inclusion/exclusion criteria);
3. If the underlying disease worsens and continued administration of the investigational drug is deemed inappropriate;
4. If complications worsen and make it difficult to continue the study;
5. If it becomes difficult to continue the study owing to adverse events;
6. If the participant is found to be pregnant;
7. If the participant exhibits extremely poor adherence, such that medication compliance is objectively deemed to be below 70%;
8. If the underlying disease is completely cured and continued administration is no longer necessary;
9. If the entire study is discontinued; and
10. If the investigator otherwise deems it appropriate to discontinue the participant’s involvement in the study for any other reason.

### Strategies to Improve Adherence to Interventions

To improve compliance, the content and importance of the protocol and the meaning of participating in the study will be explained to improve participant understanding. Furthermore, to reduce participant burden, such as by minimizing the frequency of visits, day 31 will have an option for telephone confirmation. To monitor compliance, the participants will be asked to maintain a medication diary, which will be checked regularly.

### Relevant Concomitant Care Permitted or Prohibited During the Trial

During the study period, adjustments to the administered medications or the initiation of new medications will be permitted. However, to prevent evaluation of efficacy, the use of herbal medicines with the same efficacy as orengedokuto will be prohibited. For patients at high risk of thromboembolism, antithrombotic drug (anticoagulants and antiplatelet drugs) administration should be continued where discontinuation would otherwise increase the risk of thromboembolism. Such patients include those within 2 months of coronary stent placement, those within 2 months of cerebral revascularization, those with a history of cardioembolic stroke, or those with atrial fibrillation complicated by valvular heart disease. For cases in the low-risk group for thromboembolism in which the discontinuation of antithrombotic drugs is possible, discontinuation will be considered. Once the bloody stool has stopped, antithrombotic therapy should be promptly resumed.

### Provisions for Posttrial Care

Participants will be managed by a physician according to routine care practices, including the management of any complications. The treatment protocol in this study will be covered by health insurance and will not be considered a medical procedure beyond regular medical care. Any health-related harm caused by participating in this study will be treated as appropriate according to the patient’s condition as part of regular medical care and will be covered by health insurance.

### Outcomes

#### Primary End Point

The primary end point of this study is the proportion of patients who experience rebleeding. Rebleeding is defined as the occurrence of new-onset significant hematochezia within 30 days of study enrollment (including the day of enrollment). Significant hematochezia is characterized by either a drop of 2 g/dL or more in hemoglobin level or visible gross blood that obscures the bottom of the toilet bowl. The evaluation of the outcome will be conducted by blinded investigators.

#### Secondary End Points

The secondary end points of this study are the duration of hospitalization, the number of days until the initiation of oral intake, the severity of rebleeding (including the timing and degree of anemia), and the proportion of patients experiencing recurrent rebleeding.

Safety assessments will involve evaluating each observed adverse event for its causal relationship with the investigational treatment, and the frequency of each event will be recorded accordingly.

Observational and laboratory assessment items will include detailed patient background and clinical data. These will consist of the patient identification code, the date and time of hematochezia onset, admission and discharge dates, sex, age, blood type, smoking and alcohol consumption status, history of hematochezia, history of CDB, presence of comorbidities, Charlson Comorbidity Index [24], use of nonsteroidal anti-inflammatory drugs within the past 2 weeks, concomitant medications at the time of registration, presence or absence of contrast-enhanced computed tomography, extravasation findings, and the bleeding site if present.

Other parameters to be recorded include anthropometric measurements (height and weight), general condition, vital signs, colonoscopic findings, whether bowel preparation was performed using oral lavage or enema, blood test results, medication adherence, confirmation of hematochezia, and documentation of adverse events.

### Sample Size

The target sample size was determined based on a retrospective analysis conducted at 5 participating institutions. This analysis reviewed the number of cases of CDB and the rate of rebleeding within 30 days over a 1-year period (January-December 2019; UMIN000049020). Among the patients who did not undergo endoscopic hemostasis for CDB, the 30-day rebleeding rate was 1 of 11 (9.1%) patients who received orengedokuto, compared with 15 of 48 (31.3%) patients who received only conservative treatment (*P*=.23).

The sample size was calculated based on the primary end point: the proportion of patients with rebleeding. Assuming a rebleeding rate of 31.3% in the placebo group (standard treatment only) and 9.1% in the orengedokuto treatment group, the minimum number of patients required to achieve a 2-sided significance level of 5% and a power of at least 80% was estimated. Using the Fisher exact test for a 2-sample comparison of proportions, with the PROC POWER procedure in the statistical analysis system, the required sample size was calculated to be 57 patients per group. Considering potential dropouts and ineligible cases, the planned total enrollment is set at 120 patients.

### Recruitment

At least 6 facilities will be included to ensure a sufficient sample size. Fifty-nine patients with CDB underwent conservative treatment at the 5 facilities over the course of a year; hence, we believe that by increasing the number of facilities to 6, it will be possible to achieve adequate case registration. Monthly newsletters, including recruitment rates and targets, will be provided to the investigators.

### Assignment of Interventions

#### Blinding

Prior to allocation, the person responsible for investigational drug allocation shall verify the indistinguishability of the investigational drug and placebo from each lot in terms of appearance, shape, and labeling of each treatment group.

After confirming the indistinguishability of the investigational drugs at the investigational drug storage facility, the person responsible for investigational drug allocation will assign investigational numbers to the drugs (active and placebo) in accordance with a key code table created in advance using a random number table.

The investigational drugs, labeled with drug numbers, will then be transported in fixed quantities from the storage facility to the clinical trial sites.

At the trial site, the PI (or subinvestigator) or study collaborator will log into the minimization web system provided by Medical Research Support Co, Ltd, register the study participant, and enter the allocation adjustment factors. The system will then assign the appropriate investigational drug number to each participant.

Specifications for the minimization web system are described in the allocation procedure manual and will be managed by the person responsible for drug allocation.

#### Procedure for Unblinding

In cases such as a participant’s request or the occurrence of adverse events, the investigational drug allocation manager may unblind (open the key code) and reveal the allocated intervention under the instructions of the PI and the chairperson of the Efficacy and Safety Evaluation Committee.

##### Storage of the Key Code

The key code table will be stored by the person responsible for investigational drug allocation.

##### Opening of the Key Code

The key code will be opened as follows:

1. In principle, the key code will be opened by the person responsible for drug allocation only after all drug administration is complete and the contents of the case report forms (CRFs) have been finalized.
2. The statistician will perform statistical analyses based on the key code received from the drug allocation manager.

### Investigational Drug and Placebo Management

The procedures listed below will be followed to ensure proper management and accountability of the investigational products throughout the study:

1. The receipt and return of investigational drugs will be recorded in the drug accountability record.
2. The PI or their designee will record the date of receipt and the lot number of the investigational drugs in the drug management log.
3. The investigational drugs will be stored under the recommended storage conditions (room temperature) in a secure location with restricted access.
4. During the clinical study, the following information will be recorded in the drug accountability log:
  - Patient identification code to whom the drug was dispensed; and
  - Date of dispensing, quantity dispensed, date of return, and quantity returned.
5. The drug management log will be readily available at all times for monitoring, review by the ethics committee, and inspection by other relevant regulatory authorities.
6. The drug management logs and storage locations of investigational drugs will be reviewed during monitoring.
7. Unused investigational drugs will be returned to the PI or designee as soon as they are deemed unnecessary. At the end of the study, all unused investigational drugs remaining at the trial site will be properly disposed. The monitor will confirm that disposal has been performed.

### Data Collection and Management

The PI or subinvestigator at each participating medical institution will promptly complete a CRF after collecting the specified tests and evaluation items. In this study, paper-based CRFs will be sent to the data center via fax. Data will be entered directly into a database managed by the data center.

Participants who do not attend their scheduled visits will be contacted by phone. Follow-up will be continued regardless of protocol adherence.

The PI will appoint a data management supervisor to oversee and coordinate the timely creation of CRFs and data collection to ensure the development of an appropriate dataset. The data management supervisor will be responsible for monitoring the progress of CRF completion and data entry, as well as for conducting data review and cleaning. Furthermore, the data management supervisor will be accountable for the quality of the datasets used in the analysis.

The PI will also appoint a monitoring officer to conduct central monitoring to ensure that the study is being conducted safely and in accordance with the study protocol, the Clinical Research Act, and other applicable regulations and that the data are being accurately collected. The monitoring officer will report the results to the PI.

Personal information in the database will be password-protected on a secure network. No identifying information will be used in any analysis, report, or publication. Biological specimens for genetic or molecular analysis will not be collected in this study.

### Statistical Methods

The full analysis set (FAS) will include all enrolled participants, excluding those who are found to be ineligible, those who do not receive any study treatment after enrollment, and those for whom no postenrollment measurements are available. The per-protocol set will include participants in the FAS, excluding those for whom the primary outcome is not evaluable and those with major protocol deviations, such as incorrect treatment allocation, use of prohibited concomitant treatments, or treatment adherence of 70% or less. The safety analysis set will include all enrolled participants who receive at least one dose of the study treatment.

For the primary end point, the FAS will be used as the analysis population. The proportion of patients with rebleeding will be calculated for each group, and intergroup comparisons will be performed using Fisher exact test, consistent with the method used for sample size calculation. A 2-sided significance level of 5% will be applied. Additionally, a sensitivity analysis will be conducted using the per-protocol set to assess the robustness of the results.

For the secondary end points, the FAS will also be used as the analysis population. Statistical comparisons will be performed between the 2 groups for the following variables:

1. Mean duration of hospitalization;
2. Mean number of days until the initiation of oral intake;
3. Severity of rebleeding (including timing and degree of anemia); and
4. Proportion of patients with recurrent rebleeding (re-rebleeding)

The Wilcoxon rank-sum test will be used to analyze continuous variables. Fisher exact test will be used to analyze categorical variables. No adjustment for multiplicity will be made for the secondary end point analyses, which will be considered exploratory. Further details will be prespecified in the statistical analysis plan, which will be finalized before database lock and unblinding.

The safety analysis set will be used for the safety analysis. Safety will be evaluated separately for the treatment and follow-up periods. Adverse events will be assessed in terms of type, frequency, severity, and causal relationship with the investigational drug. The incidence rates of adverse events will be calculated and compared between groups. If statistical testing is applied, the Fisher exact test will be used for comparison.

No interim analyses are planned. Missing data will not be imputed and will be treated as missing in the analysis. If missing data are expected to affect outcomes, sensitivity analyses will be planned and conducted accordingly. All sensitivity analyses will be prespecified prior to database lock.

### Oversight and Monitoring

#### Composition of the Coordinating Center and Trial Steering Committee

The management structure comprises a PI, a trial steering committee, and a data-monitoring committee. The trial steering committee will be responsible for conducting the trial and will meet regularly to discuss trial progress. The team members will include a clinical epidemiologist, a specialist in Kampo medicine, and a statistician.

The data-monitoring committee will review the interim safety data and periodically review the conduct of the study. All data will be monitored monthly. The PI shall appoint a data management supervisor who will be responsible for overseeing and coordinating the timely completion of CRFs, data collection, and the preparation of appropriate datasets. The data management supervisor will monitor the progress of CRF completion and data entry and will be responsible for data review and cleaning. Furthermore, the data management supervisor will be accountable for ensuring the quality of the analysis dataset.

#### Composition of the Data Monitoring Committee, Its Role, and Reporting Structure

The PI will appoint a data management officer to manage and coordinate the creation of CRFs and data collection without delay and the creation of an appropriate dataset. The data management officer will manage the progress of CRF creation and data entry and will be responsible for data review and cleaning. The data management officer will also be responsible for the quality of the dataset used in the analysis.

The PI will appoint a central monitor to confirm whether the study is being conducted safely and in accordance with the research protocol and the Clinical Research Act and whether data are being collected accurately. The monitor will submit the monitoring results to the PI.

#### Adverse Event Reporting and Harms

The PI or subinvestigator shall promptly provide appropriate treatment to the study participant in the event of an adverse event and document the details in the medical records. If necessary, suspension or discontinuation of the study will be considered. If treatment or other medical interventions are required, this will be explained to the participant. The name of the adverse event, its severity, causality, outcome, and other relevant details will be assessed and recorded in the CRF.

An Independent Efficacy and Safety Evaluation Committee will be established for this study. This committee will consist of experts who are independent of the study and will be responsible for assessing its progress, safety data, and key end points. The committee will provide recommendations to the PI regarding whether to continue, suspend, terminate, or revise the study protocol, as necessary.

#### Frequency and Plans for Auditing Trial Conduct

The PI shall report on the implementation status of the specified clinical study to the administrator of the implementing medical institution annually, within 2 months after each 1-year period from the date the study plan was published on the Japan Registry of Clinical Trials. Subsequently, the PI will submit a periodic report to the ethics committee specified in the study protocol.

The items to be reported on the implementation status are as follows:

1. The number of participants enrolled in the specified clinical study;
2. The occurrence and subsequent course of any diseases or conditions;
3. The occurrence of noncompliance and subsequent responses;
4. Evaluation of safety and scientific validity; and
5. Information regarding the involvement of pharmaceutical companies or other entities, as stipulated in the conflict-of-interest management standards

When the PI reports to the ethics committee, they shall promptly share the information with the other investigators. The investigators shall then promptly report the content of the information to the administrators of their respective medical institutions.

Auditing trial conduct is not planned but will be conducted as required.

#### Plans for Communicating Important Protocol Amendments to Relevant Parties

The PI will document any significant changes to the protocol and obtain approval from the review committee. Once the changes are approved, a formal notification will be sent to all PIs, co-investigators, etc. If necessary, opportunities for explanation will be established to prevent misunderstandings or lack of understanding. If the changes affect the participants, the informed consent document will be updated and reconsent will be obtained as necessary.

In addition, all change notifications and confirmation documents will be recorded in the document management ledger and maintained in a state that can be viewed during audits and monitoring.

### Secondary Use of Information

Information obtained from research participants during this study may be used for future research projects that have not yet been identified at the time that consent is obtained. In such cases, a new research protocol will be prepared, reviewed by the ethics committee, and implemented only after approval is granted.

### Dissemination Plans

The results of this study will be presented at conferences and published in scientific journals. We will also prepare lay summaries for the public and endeavor to disseminate the results more widely.

## Results

This study received funding from the Japan Agency for Medical Research and Development in May 2025. Enrollment is scheduled from July 2025 to December 2027. As of June 2026, we have enrolled 48 participants. All follow-up and data collection activities will be finalized by February 2028. Results are anticipated to be completed in the first quarter of 2028.

## Discussion

This study aims to determine whether orengedokuto can safely reduce rebleeding in patients with CDB who have achieved spontaneous hemostasis but are not candidates for endoscopic hemostasis because the source of bleeding cannot be identified. If this research hypothesis is confirmed, it will provide the first high-quality evidence supporting oral therapy for the prevention of rebleeding in this patient population, which is clinically challenging to manage.

Endoscopic therapy is recommended in cases of active bleeding or SRH. A multicenter retrospective study involving 5823 cases of CDB reported that both early and late recurrent bleeding rates were significantly lower in patients with definitive CDB who received endoscopic treatment than in those with presumptive CDB who were managed conservatively [25]. Moreover, the study showed that the endoscopic treatment of SRH significantly reduced early recurrent bleeding rates compared with untreated SRH (17.4% vs 26.7%, *P*=.04), highlighting the importance of actively identifying SRH.

However, active bleeding is often not observed during colonoscopy, and the presence of multiple diverticula makes the identification of the bleeding source difficult, resulting in a relatively low detection rate. As a result, despite the risk of rebleeding, many patients are managed with conservative treatment. Currently, there is no evidence supporting the efficacy of pharmacological therapy in achieving hemostasis when endoscopic intervention is not feasible. Consequently, patients often require repeated blood transfusions and frequent endoscopic examinations owing to recurrent bleeding, which not only reduces their quality of life but also imposes a significant economic burden. This study was designed to address this critical lack of evidence.

Orengedokuto (Huanglian Jiedu Tang) is a traditional Kampo medicine. In addition to its well-documented clinical applications in traditional medicine, a growing body of modern research has focused on elucidating the pharmacological mechanisms of orengedokuto at the molecular level. Orengedokuto has been suggested to contribute to primary hemostasis through mechanisms such as vasoconstriction of small vessels and suppression of excessive activation of the coagulation-fibrinolysis system. Several studies have demonstrated its ability to suppress oxidative stress by reducing the formation of reactive oxygen species and enhancing endogenous antioxidant systems [26]. This activity is considered to be one of the fundamental mechanisms underlying its therapeutic effects in managing chronic inflammatory conditions, tissue injury, and metabolic disorders. Furthermore, under the administration of nonsteroidal anti-inflammatory drugs, orengedokuto has been suggested to exert mucosal protective effects by increasing prostaglandin E2 production [27] and elevating adenosine levels by suppressing adenosine deaminase expression [28].

In our retrospective study of patients with CDB who did not undergo endoscopic hemostasis, the rebleeding rate was lower in the orengedokuto group than in the conservative treatment group, although the difference was not statistically significant. Based on these findings, we initiated a double-blind randomized controlled trial to evaluate the efficacy and safety of orengedokuto in preventing rebleeding in patients with CDB of unknown origin. This study has several strengths. To the best of our knowledge, this is the first multicenter, double-blind, randomized, placebo-controlled trial to evaluate the efficacy of Kampo medicine for the prevention of gastrointestinal bleeding. This study specifically targets patients for whom no established preventive therapy currently exists, thereby addressing an unmet clinical need. However, it is important to recognize some limitations. First, the sample size calculation is based on a very small retrospective cohort of just 11 patients in the orengedokuto group. The assumed absolute risk reduction of around 22% could lead to an overestimation of the true treatment effect. Since this is an exploratory study, the sample size is relatively small; if efficacy is demonstrated, the results will need to be confirmed in a larger confirmatory study. Furthermore, because the follow-up period was limited to 30 days, it was not possible to evaluate long-term rebleeding or late adverse events. Future studies with a longer follow-up period are needed to confirm the durability of the treatment effect and to evaluate potential late complications.

If this study demonstrates that the frequency of rebleeding in patients with diverticular bleeding who cannot be treated by colonoscopy can be reduced, patients’ quality of life may improve and medical costs will decrease. Furthermore, our findings may be incorporated into treatment guidelines for diverticular bleeding.

## Supplementary material

10.2196/93449 Checklist 1 SPIRIT 2025 checklist.

## Abbreviations

ALGIB: acute lower gastrointestinal bleeding
CDB: colonic diverticular bleeding
CRF: case report form
FAS: full analysis set
PI: principal investigator
SPIRIT: Standard Protocol Items: Recommendations for Interventional Trials
SRH: stigmata of recent hemorrhage
